# 18F-Fluorodeoxyglucose Positron Emission Tomography–Computed Tomography Findings of Polymyalgia Rheumatica in Patients with Giant Cell Arteritis

**DOI:** 10.3390/jcm12226983

**Published:** 2023-11-08

**Authors:** Elena Heras-Recuero, Marta Martínez de Bourio-Allona, Laura Cristina Landaeta-Kancev, Teresa Blázquez-Sánchez, Arantxa Torres-Roselló, Miguel Álvarez-Rubio, Mariam Belhaj-Gandar, Juan Antonio Martínez-López, Luis Martínez-Dhier, Javier Llorca, Raquel Largo, Miguel Ángel González-Gay

**Affiliations:** 1 Division of Rheumatology, ISS-Jiménez Díaz Foundation University Hospital, 28040 Madrid, Spain; elena.herasr@fjd.es (E.H.-R.); teresa.blazquezs@quironsalud.es (T.B.-S.); arantxa.torres@quironsalud.es (A.T.-R.); miguel.arubio@quironsalud.es (M.Á.-R.); mariam.bgandar@quironsalud.es (M.B.-G.); jamartinez@quironsalud.es (J.A.M.-L.); rlargo@fjd.es (R.L.); 2 Department of Nuclear Medicine, Fundación Jiménez Díaz University Hospital, 28040 Madrid, Spain; marta.martinezb@quironsalud.es (M.M.d.B.-A.); laura.landaeta@quironsalud.es (L.C.L.-K.); lmartinez@quironsalud.es (L.M.-D.); 3 CIBER Epidemiología y Salud Pública (CIBERESP), Department of Medical and Surgical Sciences, University of Cantabria, 39011 Santander, Spain; javierllorca1958@gmail.com; 4 Medicine and Psychiatry Department, University of Cantabria, 39008 Santander, Spain

**Keywords:** giant cell arteritis, polymyalgia rheumatica, positron emission tomography–computed tomography (PET-CT) with 18F-fluorodeoxyglucose (FDG), FDG uptake, large vessel vasculitis

## Abstract

Objective: Giant cell arteritis (GCA) and polymyalgia rheumatica (PMR) are often overlapping conditions. We studied whether 18F-fluorodeoxyglucose (FDG) positron emission tomography–computed tomography (PET-CT) is useful in identifying PMR in the setting of large vessel (LV) GCA. Methods: LV-GCA patients diagnosed by PET-CT at a tertiary care center for a population of 450,000 people over a two-year period were reviewed. Scoring was performed based on potential significant FDG uptake at up to 16 sites in nine different extravascular areas (SCORE 16). Differences in extravascular sites of significant FDG uptake were evaluated between LV-GCA with a clinical diagnosis of PMR or not. Results: Fifty-four patients were diagnosed with LV-GCA by 18F-FDG-PET-CT. Of them, 21 (38.8%) were clinically diagnosed with PMR. Significant extravascular FDG uptake was more frequently observed in those with a clinical diagnosis of PMR. In this sense, the SCORE 16 was higher in those with clinical PMR (5.10 ± 4.05 versus 1.73 ± 2.31 in those without a clinical diagnosis of PMR; *p* < 0.001). A SCORE 16 involving more than four sites of significant FDG uptake yielded a sensitivity of 52% and a specificity of 91% for establishing a clinical diagnosis of PMR associated with LV-GCA. The best areas of significant FDG uptake to clinically identify PMR in patients with LV-GCA were the shoulder, the greater trochanter, and the lumbar interspinous regions, with an area under the ROC curve of 0.810 (0.691–0.930). Conclusions: Significant extravascular 18F-FDG-PET-CT uptake may help establish a clinical diagnosis of PMR in patients with LV-GCA. These patients are more commonly diagnosed with PMR if they have significant FDG uptake in the shoulder, greater trochanter, and lumbar interspinous areas.

## 1. Introduction

Giant cell arteritis (GCA) and polymyalgia rheumatica (PMR) are entities frequently observed in individuals older than 50 years from Western countries [1,2]. Patients with PMR have pain and stiffness involving bilaterally the shoulder girdle, proximal aspects of the arms, the neck, the hip girdle, and proximal aspects of the thighs [3]. GCA is a large vessel vasculitis (LVV) that in the classic pattern of the disease affects predominantly extracranial arteries derived from the carotid. In this sense, vascular inflammation due to the involvement of arteries derived from the external carotid artery leads to typical manifestations of the disease, such as headache, scalp tenderness, jaw claudication or dysphagia, while the involvement of branches of the internal carotid promotes the development of the feared visual ischemic manifestations of the disease [4]. The advent of new imaging techniques has been a great step forward for the diagnosis of GCA [5]. With respect to this, the use of positron emission tomography–computed tomography (PET-CT) with 18F-fluorodeoxyglucose (18F-FDG) enables the identification of GCA patients with a predominantly extracranial LV vasculitis pattern of the disease [6]. This functional imaging technique combines 18F-FDG-PET-CT which allows for the identification of LV vasculitis due to its capacity to show glucose uptake from the high activity of inflammatory cells in the vessel walls. In this regard, PET imaging shows increased vascular FDG uptake in more than 80% of patients with GCA. 18F-FDG-PET-CT has proven to be very useful in detecting the presence of vascular inflammation of the arteries of the lower extremities in patients with GCA [7,8]. Moreover, 18F-FDG-PET-CT may help to rule out other entities such as infection or malignancy [9,10].

During the last 25 years, different researchers, in particular the group of Cantini et al., have confirmed that magnetic resonance imaging and, in particular, ultrasonography are very useful for the diagnosis of PMR [11,12,13,14]. Due to this, experts who elaborated the 2012 provisional European League Against Rheumatism/American College of Rheumatology classification criteria for PMR included the presence of an ultrasonography showing shoulder bursitis and/or biceps tenosynovitis and/or glenohumeral synovitis and hip with synovitis and/or trochanteric bursitis as a criterion for the presence of PMR [15]. Although 18F-FDG-PET-CT is not included in these proposed classification criteria for the diagnosis of PMR, several studies support the use of 18F-FDG-PET-CT for the diagnosis of PMR [16,17]. On this basis, van der Geest et al. performed a meta-analysis that included a systemic review of 636 patients from nine studies. They concluded that 18F-FDG-PET-CT can be useful in establishing a diagnosis of PMR when it is suspected [18]. Interestingly, studies with 18F-FDG-PET-CT have demonstrated the presence of underlying LV vasculitis in 14–40% of patients with PMR [17,18,19,20]. This frequency of underlying LV vasculitis may be higher in patients with PMR who present with inflammatory lower back pain, severe pain in the pelvic girdle, and a presence of diffuse pain in the lower extremities [21].

An issue which from our point of view has been less evaluated is the usefulness of 18F-FDG-PET-CT to identify PMR in patients diagnosed with LV-GCA.

To address this question and determine the best predictors of 18F-FDG-PET-CT to clinically identify PMR among individuals with LV-GCA, we evaluated a series of patients diagnosed with LV-GCA by 18F-FDG-PET-CT.

The results provided in the present study may be unique because these 18F-FDG PET-CT studies in patients with LV-GCA rarely focus on PMR findings on imaging since GCA will be the primary diagnosis and the patient will be treated accordingly due to presence of this vasculitis. However, in addition to proper clinical diagnosis, patients with LV-GCA are known to more commonly have PMR and more disease relapses than those with the typical cranial phenotype of GCA. In this context, it is possible that the presence of PMR helps us to identify a subgroup of GCA patients who are more likely to develop relapsing disease.

## 2. Patients and Methods

### 2.1. Study Design and Patient Recruitment

A retrospective study was conducted on patients undergoing 18F-FDG-PET-CT at the Fundación Jiménez Díaz University Hospital (Madrid, Spain) between April 2021 and March 2023. As reported, 54 of them were diagnosed with LV-GCA using 18F-FDG-PET-CT [22]. These 54 patients were evaluated for the presence of clinical and 18F-FDG-PET-CT features of PMR.

The study procedures followed the recommendations of the 1975 Declaration of Helsinki, revised in 2000. Ethical committee approval was obtained (PIC034-23).

First, we identified those patients who were clinically diagnosed with PMR in the context of LV-GCA by physicians with high experience in diagnosing PMR. We then analyzed possible differences in sites with significant extravascular FDG uptake between patients clinically diagnosed with PMR or not. Second, we evaluated the cut-off points for the diagnosis of PMR according to the sites where FDG uptake was significant. In a third step, we evaluated the best areas of significant FDG uptake to clinically identify the presence of clinical PMR in patients with LV-GCA.

### 2.2. Study Protocol

#### 2.2.1. Patient Disease Assessment

Fundación Jiménez Díaz University Hospital provides medical care to 450,000 people. Between April 2021 and March 2023, 1302 patients underwent PET-CT. As previously described [22], the Big Data Department of the Jiménez Díaz Foundation Hospital conducted a search for patients who underwent PET-CT during the two years of the study. In a further step, the 18F-PET-CTs of patients that included any of the following keywords: “vasculitis”, “large vessels”, “medium vessels”, “vascular wall”, “vascular”, “aortitis”, “vessel inflammation”, “increased FDG vessel uptake”, “polymyalgia rheumatica”, and “giant cell arteritis” were evaluated by Rheumatology and Nuclear Medicine physicians. The patients included in the study were those whom agreement regarding the presence of LV-GCA was confirmed.

At the time of diagnosis of LV-GCA, physicians with experience in autoimmune diseases had made a clinical diagnosis of PMR if patients had new-onset symmetrical pain and stiffness in the shoulder girdle and proximal arms, often associated with pain and stiffness in the pelvic girdle, hips, proximal aspects of the lower extremities, and neck. Regarding this, the clinical diagnosis of PMR was made independently of the 18F-FDG-PET-CT data. All patients were seen by physicians who ordered 18F-FDG-PET-CT based on the information obtained in the clinical evaluation. Clinical information was obtained by reviewing medical records.

#### 2.2.2. FDG-PET-CT Equipment, Protocol, and Interpretation

PET-CT examinations were carried out using an integrated digital PET-CT system (GE Discovery MI3R, with NEMA sensitivity of 7.5 cps/kbq, 3 rings and 15 cm axial field of view). Patients received 175–350 mbq (2.5–5.0 MBq/kg) of 18F-FDG after at least a 4 h fast. Subsequently, a post injection rest time of 60 min was carried out. The PET-CT study was brought about in a supine position, with the arms stretched above the head. Scans were obtained from the base of the skull to the femur. Low-dose CT was performed for PET co-registration (140 kv, 380 ma) and was followed by PET imaging (1.45 min per bed). In all patients, the blood glucose levels before tracer injection were <200 mg/mL.

18F-PET-CT scans were evaluated by experienced nuclear medicine physicians. They evaluated the distribution of the radiopharmaceutical in the aorta carotids, brachiocephalic trunk, subclavian, axillary, vertebral, humeral, iliac, and femoral arteries using a visual uptake classification scale as recently described [22]. In this regard, the standardized grading system ranged from 0 to 3 (vascular to hepatic uptake): following this procedure, the values obtained ranged from 0 = which meant no uptake (≤mediastinum); 1 = considered as low-grade uptake (<liver); 2 = intermediate-grade uptake (=liver), and 3 = defined as high-grade uptake (>liver). Following this procedure, both grade 2 (considered as indicative) and grade 3 (considered as strongly) were estimated to be positive for the presence of LV vasculitis. Moreover, reevaluation of the positive 18F-PET-CT scans was conducted to confirm the results [22].

A similar procedure was conducted to assess the sites of significant extravascular FDG uptake. With respect to this, extravascular sites of FDG uptake were evaluated using a semiquantitative analysis as previously described [23,24]. This assessment was graded as follows; Grade 0 = absence of uptake; Grade 1: uptake less than the liver uptake; Grade 2: uptake similar to the liver uptake, and Grade 3: uptake higher than the liver uptake. The score was established by members of the Nuclear Medicine Department. They performed the analysis as follows: For each skeletal extravascular region, scores < 2 were considered to be negative, while scores ≥ 2 were considered positive. Therefore, a site was considered as having significant extravascular FDG uptake if the score was ≥2.

We assessed 9 extravascular areas and a total of 16 sites of potential involvement: two acromioclavicular and sternoclavicular joints, two hips, two shoulders, two greater trochanters, two ischial tuberosities, two symphysis pubis enthesis, and the cervical and lumbar interspinous processes. We calculated a total score (0–16), corresponding to the sum of all sites with significant FDG uptake, for each patient. This score is called SCORE 16 throughout the manuscript.

At the time of the study, these sites of significant FDG uptake were again reviewed for confirmation.

### 2.3. Statistical Analysis

Fisher’s exact test (two-tailed) for a 2 × 2 contingency table was used to establish whether a significant association existed between two categorical variables. A logistic regression penalized via LASSO was carried out to identify the best areas of significant extravascular FDG uptake to identify clinical PMR. LASSO penalization used all 9 areas assessed and iteratively shrank their coefficients to 0 (i.e., delete the location from the model) to obtain a model with a small number of predictors. The criterion for shrinking coefficients was obtained via cross-validation. The ability of SCORE 16 and the logistic regression model to classify patients with clinical PMR was measured with the area under the ROC curve. The best cut off was determined using the Youden index. Statistical analyses were performed using the Stata 18/SE (StataCorp, College Station, TX, USA) software.

## 3. Results

### 3.1. Differences in Significant Extravascular Sites of FDG Uptake between Patients with LV-GCA Clinically Diagnosed with PMR or Not

During the period of study, 21 of the 54 patients who were diagnosed with LV-GCA by 18F-FDG-PET-CT were also diagnosed clinically with PMR.

All PMR patients included in this study were over 50 years old and complained of pain at night and stiffness that was worse in the morning. All but six had an erythrocyte sedimentation rate (ESR) greater than 40 mm/1st hour and/or a C-reactive protein (CRP) greater than 10 mg/L at the time of disease diagnosis of LV-GCA and fulfilled the 2012 European League Against Rheumatism/American College of Rheumatology provisional classification criteria for PMR [15].

As previously reported in a small proportion of patients with PMR [25], the six PMR patients with low or normal ESR and CRP had typical inflammatory pain involving the arms and shoulders. In this regard, Cantini et al. described that PMR patients with normal ESR have similar bilateral subacromial bursitis involvement to those with high ESR when magnetic resonance imaging or ultrasonography studies are performed [26]. Moreover, other conditions mimicking PMR including other rheumatic diseases or malignancies [27,28] were excluded at diagnosis or during follow-up.

The differences in the presence of significant extravascular uptake in the sixteen sites evaluated between patients with PMR or not are described in Table 1.

**Figure 1:**
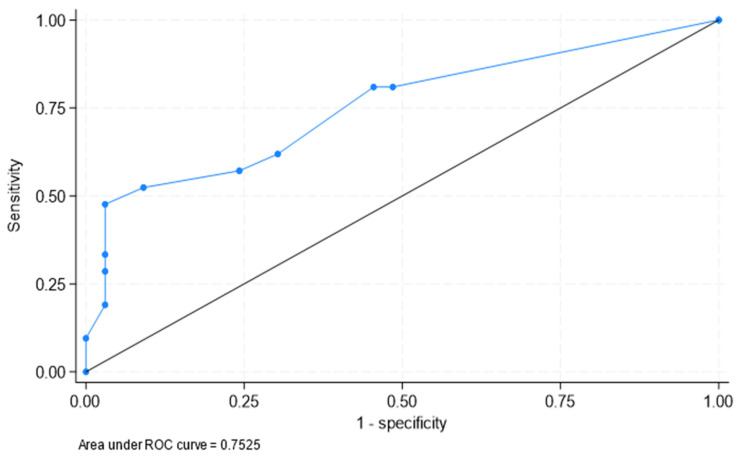
ROC curve on the diagnostic capacity of SCORE 16 to identify clinical PMR. The area under the ROC curve was 0.753 (95% confidence interval [CI]: 0.615, 0.890).

Patients with a clinical diagnosis of PMR more commonly had significant extravascular FDG uptake in the sites explored. This was especially true when comparisons of significant FDG uptake in the shoulders were assessed (76.2% in the patients who were diagnosed clinically with PMR versus 24.2% in those without PMR; *p* < 0.001). Other sites such as cervical and lumbar interspinous processes, the hip, and the greater trochanter also more commonly showed significant FDG uptake in those with a clinical diagnosis of PMR (*p* value for all the comparisons < 0.05). Differences in significant FDG uptake in other sites are shown in Table 1. Consequently, the SCORE 16 of significant FDG uptake was higher in patients with LV-GCA and PMR than in those without PMR (5.10 ± 4.05 versus 1.73 ± 2.31; *p* < 0.001). According to these results, the number of affected extravascular sites was greater in patients with PMR. Figure 1 shows the ROC curve on the diagnostic capacity of SCORE 16 to identify clinical PMR.

### 3.2. Determination of the Best Cut-Offs for the Diagnosis of PMR in Patients with LV-GCA

Based on the number of sites of significant FDG uptake, an ROC curve analysis was performed to determine the optimal cut-offs to clinically identify PMR in patients with LV-GCA. The results of the cut-off with the best Youden index were the following: more than five (sensitivity 47.6, specificity 97%); more than four (sensitivity 52%, specificity 91%); and more than three (sensitivity 57%, specificity 76).

Taking into account these results, a model that included patients with SCORE 16 > 4 would be 52% sensitive and 91% specific for clinical diagnosis of PMR. However, a lower cut off of SCORE 16 would decrease the specificity to clinically identify PMR. In this regard, the inclusion of patients with SCORE 16 > 3 would be 57% sensitive and 76% specific.

### 3.3. Best Areas of Significant FDG Uptake to Clinically Identify PMR in Patients with LV-GCA

**Figure 2:**
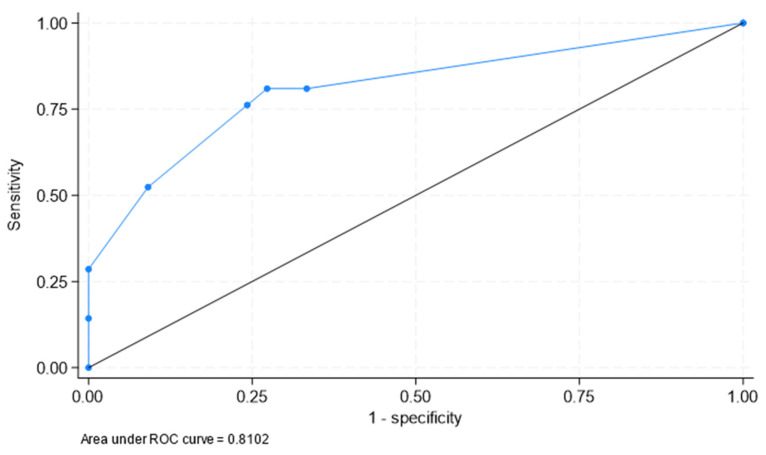
ROC curve on the ability of a model with three locations (shoulder, greater trochanter, and lumbar interspinous) to identify clinical PMR. Based on that, the area under the ROC curve was 0.810 (95% CI: 0.691, 0.930).

A logistic regression penalized via LASSO was carried out to identify the best areas of significant FDG to clinically identify PMR in patients with LV-GCA. The final model included only three locations: shoulder (odds ratio [OR] = 8.6, 95% CI: 2.0, 36.4), greater trochanter (OR = 4.4, 95% CI: 0.8, 26.1), and lumbar interspinous (OR = 1.6, 95% CI: 0.3, 7.9), with an area under the ROC curve = 0.810 (95% CI: 0.691, 0.930) (Figure 2).

Therefore, patients with LV-GCA were more commonly diagnosed with PMR if they had significant FDG uptake in the shoulder, the greater trochanter, and the lumbar interspinous areas.

## 4. Discussion

The present study shows that in patients with LV-GCA, a significant uptake of FDG in some extravascular anatomical areas helps to establish a clinical diagnosis of PMR. This is especially true when the shoulder, greater trochanter, and lumbar interspinous areas are affected. These findings are consistent with previously reported information on 18F-FDG-PET-CT in PMR. In this regard, in a systematic review of the literature, van der Geest et al. highlighted that the shoulders, sternoclavicular joints, interspinous bursae, ischial tuberosities, hips, and greater trochanters are relevant anatomical areas that should be evaluated by 18F-FDG-PET-CT in patients with suspected PMR [18]. They also concluded that significant FDG uptake at a combination of anatomical sites provides useful information for making a diagnosis of PMR [18].

Casadepax-Soulet et al. recently performed a retrospective study in 85 patients with new-onset PMR and 75 controls who underwent 18F-FDG PET-CT [24]. In addition to a quantitative analysis of FDG uptake, they also performed a semiquantitative analysis using the same methodology applied in our report. In this sense, they analyzed the significant absorption of FDG in sixteen sites, corresponding to the nine areas studied in our work. Patients who had recent onset of PMR showed a significantly higher mean number of sites with significant FDG uptake compared to controls [24]. The most common areas of significant FDG uptake were the hips, shoulders, and ischial tuberosity, with 89%, 88%, and 88% significant FDG uptake, respectively. Although the location of hypermetabolic involvement was not very different from that found in our study, the frequency of increased FDG uptake was greater than in our series of PMR associated with LV-GCA. The smaller number of patients included in our study and the different type of patients, since they basically evaluated patients with an initial diagnosis of PMR while we analyzed patients who in all cases presented LV-GCA, may explain these differences.

Shoulder involvement is the hallmark of PMR. This area showed the highest frequency of significant FDG uptake (76.2%) in our series. This was greater than in the series of 50 patients with PMR described by Sondag et al. (58%) [16] but more reduced than that described by Henckaerts et al. and Yamashita et al. who studied 99 consecutive individuals with a possible diagnosis of PMR and 14 untreated patients with active PMR, respectively [17,29].

In keeping with Casadepax-Soulet et al., who described in their PMR series a low frequency of significant FDG uptake in the acromioclavicular joints compared to other areas [24], patients with PMR associated with LV-GCA in our series had a lower frequency of significant FDG uptake in acromioclavicular and sternoclavicular involvement. Furthermore, we observed no statistically significant differences in hypermetabolic FDG uptake in these areas when comparing LV-GCA with PMR to those without PMR.

Several studies showed a strong correlation between cervical interspinous bursitis manifested as high-contrast enhancement magnetic resonance imaging and high 18F-FDG-PET-CT uptake in patients with PMR [4,29]. A strong association between interspinous bursitis in the lumbar spine with PMR was also disclosed using MRI [30]. In this regard, an increased FDG uptake in the interspinous process seems to have high specificity for PMR [20,31]. This was also the case in our series of PMR associated with LV-GCA. In this sense, the presence of significant FDG uptake in the lumbar interspinous process was one of the predictors of PMR in LV-GCA found in our study.

In our study, we also aimed to establish the best cut-off point for the diagnosis of PMR in patients with LV-GCA. We found that a model including patients with more than four sites of significant FDG uptake produced high specificity (91%) but low sensitivity (52%) for a clinical diagnosis of PMR in patients with LV-GCA. In contrast, Casadepax-Soulet et al. achieved much better sensitivity and specificity. They described that ≥5 hypermetabolic sites provided 96.5% of sensitivity and 100% of specificity for the diagnosis of PMR [24]. Other studies have described a lower sensitivity and specificity for the diagnosis of PMR and these differences may be explained by the different definitions of significant FDG uptake and the different scoring system, as well as the variable use of glucocorticoids before evaluation with 18F-FDG -PET-CT. However, although we were unable to achieve robust cutoffs for diagnosing PMR in patients with LV-GCA, we observed that a model with three locations (shoulder, greater trochanter, and lumbar interspinous) was very useful in identifying clinical PMR in LVV-GCA patients. Of note, Flaus et al. established a decision tree with only two extravascular musculoskeletal sites to make a diagnosis of PMR by 18F-FDG PET-CT [32]. These authors used machine learning to define a brief decision tree capable of detecting patients with PMR in a large retrospective cohort composed of 55 patients with PMR and 85 patients with other inflammatory rheumatic diseases. Machine learning facilitated increasing the diagnostic value of visually assessed musculoskeletal sites. Splitting rules of the decision tree were established on the presence of a positive interspinous bursa or, if negative, a positive trochanteric bursa. Based on the decision tree, the sensitivity and specificity for diagnosing PMR were 73.2% and 87.5% in the training cohort and 78.6% and 80.1% in the validation cohort [32]. In agreement with these results, besides shoulders, greater trochanter and lumbar interspinous process were the best areas of significant FDG to clinically identify PMR in patients with LV-GCA in our study.

As mentioned above, an important difference between our study and most reports on 18F-FDG-PET-CT in PMR was that all of our patients had been diagnosed with LV-GCA. In this sense, classic studies highlighted the frequent overlap between PMR and GCA [33]. This fact has recently been discussed in depth by Salvarani et al. [34]. Patients with isolated PMR have some clinical differences compared to PMR patients with biopsy-proven GCA who exhibit the typical cranial pattern of the disease. In comparison, patients with isolated PMR were found to be younger, with a lower frequency of constitutional manifestations and a less severe inflammatory response manifested by lower ESR values, higher hemoglobin levels, and lower platelet counts than patients with PMR associated with biopsy-proven GCA [35]. This does not appear to be the case when silent LV-GCA is diagnosed in patients presenting as isolated PMR. In this regard, patients who have a predominant extracranial LV-GCA disease pattern tend to be younger, have a longer duration of symptoms before the LV-GCA diagnosis is made, more commonly exhibit disease relapses, less commonly have a positive temporal artery biopsy, and may require a longer duration of therapy [36]. Furthermore, these patients more frequently have features of PMR than those with the predominantly cranial pattern of GCA [34,36,37]. This fact may indicate that in some cases PMR may be an alarm sign of the presence of an underlying LV-GCA [34,37]. In this sense, Salvarani et al. noted that LV-GCA is observed in at least 22–25% of patients presenting with isolated PMR [34]. This high number of patients with silent LV-GCA raises the question of whether we should routinely perform an imaging technique to identify underlying LV-GCA in patients with PMR. Certainly, early identification of LV-GCA can help prevent vascular complications such as aortic aneurysms and dissection and arterial stenosis [38]. Moreover, 18F-FDG-PET-CT performed at the time of LV-GCA diagnosis can help estimate the risk of aortic aneurysm formation [39]. With respect to this, we recently proposed that imaging techniques should be used to identify an underlying LV-GCA in patients with PMR who do not have the typical cranial ischemic manifestations of GCA if they present with inflammatory low back pain, severe manifestations involving the pelvic girdle, bilateral diffuse pain in the lower extremities, presence of a high inflammatory response manifested by an ESR greater than 80–90 mm/1st hour, thrombocytosis or a hemoglobin lower than 11 g/dL, or an incomplete response to 20 mg/day of prednisone [40].

Since 18F-FDG-PET-CT remains an expensive technique, we recently suggested that Doppler ultrasound and/or a temporal artery biopsy should be the first procedures to be performed to confirm a diagnosis of GCA in typical patients who present with cranial arteritis manifestations [22]. Because of that, in clinical practice we perform 18F-FDG-PET-CT when we suspect GCA and cranial arteritis manifestations are not relevant. In keeping with this, Desvages et al. pointed out that the use of 18F-FDG-PET/CT should not be routinely recommended in typical cases of PMR [41]. These authors recommend performing 18F-FDG-PET-CT if patients with PMR present some atypical features, laboratory markers of inflammation are not elevated, or if a history of cancer is present. Nevertheless, these authors confirmed that 18F-FDG-PET-CT can be an important diagnostic tool in patients with suspected PMR [42].

Recently, Van der Geest et al. compared well-established FDG-PET-CT scores for PMR. To do this, they evaluated 39 consecutive patients diagnosed with PMR and 19 PMR comparators. Concomitant LV-GCA was present in 10 (26%) of 39 patients with PMR. Consistent with our findings, the presence of LV-GCA was associated with lower 18F-FDG-PET-CT scores in patients with PMR [42]. However, a possible explanation for the lower frequency of PMR-related FDG uptake in our series of patients with LV-GCA may be that our patients with GCA were evaluated earlier in their disease process for PMR than in other series of patients presenting with isolated PMR. So, perhaps PMR symptoms would not have been recognized or investigated if these patients had not also presented with LV-GCA.

Alternatively, it is possible that another reason for the low sensitivity found in our study is that some patients with LV-GCA without PMR also have some subclinical PMR uptake. However, if this had been the case, the specificity but not the sensitivity would have decreased.

To analyze our data in the context of previous scores, based on the information included in an interesting study reported by Van der Geest et al. [42], we evaluated our data using the Leuven score. In this case, we obtained an area under the ROC curve = 0.7518, 95% CI: 0.6123, 0.8914. Using the prespecified cutoff of 16, the sensitivity was 33.3% and the specificity was 97.0%. Likewise, when we applied the Modified Leuven score, we obtained an area under the ROC curve = 0.6789, 95% CI: 0.5257, 0.8322. Using the prespecified cutoff of eight, the sensitivity was 42.9% and specificity 93.9%. Therefore, in both cases, the sensitivity obtained in our study to identify PMR in patients with LV-GCA was very low.

We realize that our study has several limitations, mainly due to its retrospective nature and the number of patients included in the assessment. Unlike patients with LV-GCA without cranial ischemic manifestations who, in many cases (26 of 41), had not received glucocorticoids before the PET-CT examination, 10 of the 13 patients who had cranial ischemic symptoms began glucocorticoid treatment within the week prior to the PET-CT evaluation. Therefore, the use of glucocorticoids was a limitation as it may influence the results by reducing the sensitivity of 18F-FDG-PET-CT to identify areas of significant extravascular uptake. In this regard, Nielsen et al. highlighted that 3 days of high-dose glucocorticoid therapy attenuates 18F-FDG uptake in LV-GCA [43]. Furthermore, another possible limitation was due to the fact that we could not compare our data with a subgroup of patients with isolated PMR because 18F-FDG-PET-CT was not performed in patients presenting with typical isolated PMR at our center. Finally, another limitation of our study was related to the way significant extravascular uptake was assessed in our study. In this regard, we dichotomized the score into <2 and ≥2 rather than including a visual score. Therefore, we did not evaluate the areas using a visual score (0–3). We believe that an analysis that included an uptake score of 0–2, as described by Van der Geest et al. [42], would likely have produced a higher sensitivity for identifying PMR in our patients with LV-GCA.

However, it also has strengths mainly due to the inclusion of consecutive patients with LV-GCA who were evaluated homogeneously and the exhaustive study of the images by experts in nuclear medicine. Furthermore, unlike most studies that addressed 18F-FDG-PET-CT in PMR, we studied the role of 18F-FDG-PET-CT in identifying PMR in patients diagnosed with LV-GCA.

In conclusion, although the sensitivity of 18F-FDG-PET-CT for PMR was quite low in our population, significant extravascular 18F-FDG-PET-CT uptake may help establish a clinical diagnosis of PMR in patients with LV-GCA. These patients are most commonly diagnosed with PMR if they have significant FDG uptake in the shoulder, greater trochanter, and lumbar interspinous areas.

## 5. Significance and Innovation

- 18F-FDG-PET-CT can be useful to identify PMR in patients presenting with LV-GCA.
- In patients with LV-GCA, the presence of significant 18F-FDG-PET-CT uptake in the shoulder, greater trochanter, and lumbar interspinous areas allows PMR to be identified.

## Figures and Tables

**Table 1:** Extravascular 18F-FDG PET-CT findings in 54 patients with LV-GCA according to the presence of a clinical diagnosis of PMR or not.

Extravascular Area	Site	No Clinically Evident PMR (N = 33)	Clinically Evident PMR (N = 21)	*p*
Acromioclavicular	Total	2 (6.1)	3 (14.3)	0.37
	Left	2 (6.1)	2 (9.5)	0.64
	Right	1 (3.0)	3 (14.3)	0.29
Sternoclavicular	Total	7 (21.2)	6 (28.6)	0.75
	Left	7 (21.2)	6 (28.6)	0.75
	Right	7 (21.2)	6 (28.6)	0.75
Shoulder	Total	8 (24.2)	16 (76.2)	<0.001
	Left	8 (24.2)	16 (76.2)	<0.001
	Right	7 (21.2)	14 (66.7)	0.001
Cervical interspinous		1 (3.0)	5 (23.8)	0.03
Lumbar interspinous		4 (12.1)	9 (42.9)	0.02
Hip	Total	6 (18.2)	10 (47.6)	0.03
	Left	6 (18.2)	10 (47.6)	0.03
	Right	5 (15.2)	10 (47.6)	0.01
Ischial tuberosity	Total	2 (6.1)	5 (23.8)	0.10
	Left	1 (3.0)	5 (23.8)	0.03
	Right	2 (6.1)	5 (23.8)	0.10
Greater trochanter	Total	3 (9.1)	7 (33.3)	0.04
	Left	3 (9.1)	6 (28.6)	0.13
	Right	3 (9.1)	7 (33.3)	0.04
Symphysis pubis	Total	0 (0.0)	1 (4.8)	0.39
	Left	0 (0.0)	1 (4.8)	0.39
	Right	0 (0.0)	1 (4.8)	0.39
Number of sites involved (SCORE016)	(mean ± SD)	1.73 ± 2.31	5.10 ± 4.05	<0.001
	Area under ROC curve = 0.753 (0.615, 0.890)			

## Data Availability

The data presented in this study are available on request from the corresponding author.
